# The evolution of asymmetric genitalia in Coleoptera

**DOI:** 10.1098/rstb.2015.0400

**Published:** 2016-12-19

**Authors:** Menno Schilthuizen, Paulien de Jong, Rick van Beek, Tamara Hoogenboom, Melanie Meijer zu Schlochtern

**Affiliations:** 1 Endless Forms Group, Naturalis Biodiversity Center, Darwinweg 2, 2333CR Leiden, The Netherlands; 2 Institute for Biology Leiden (IBL), Sylviusweg 72, 2333BE Leiden, The Netherlands; 3 Faculty of Earth and Life Sciences, VU University Amsterdam, De Boelelaan 1085-1087, 1081 HV Amsterdam, The Netherlands

**Keywords:** chirality, beetles, penis, sexual selection

## Abstract

The evolution of asymmetry in male genitalia is a pervasive and recurrent phenomenon across almost the entire animal kingdom. Although in some taxa the asymmetry may be a response to the evolution of one-sided, male-above copulation from a more ancestral female-above condition, in other taxa, such as Mammalia and Coleoptera, this explanation appears insufficient. We carried out an informal assessment of genital asymmetry across the Coleoptera and found that male genital asymmetry is present in 43% of all beetle families, and at all within-family taxonomic levels. In the most diverse group, Cucujiformia, however, genital asymmetry is comparatively rare. We also reconstructed the phylogeny of the leiodid tribe Cholevini, and mapped aspects of genital asymmetry on the tree, revealing that endophallus sclerites, endophallus, median lobe and parameres are, in a nested fashion, increasingly unlikely to have evolved asymmetry. We interpret these results in the light of cryptic female choice versus sexually antagonistic coevolution and advocate further ways in which the phenomenon may be better understood.

This article is part of the themed issue ‘Provocative questions in left–right asymmetry’.

## 1. Introduction

Bilateral asymmetry of male genitalia occurs in many animal groups with internal fertilization [1,2], and is especially common in the Platyhelminthes, Arthropoda, Chordata and Nematoda [3]. Its taxonomic distribution is, however, seemingly erratic. Genital asymmetry may be a defining character of clades of any size, ranging from entire orders down to individual species, suggesting numerous (probably thousands) events of parallel evolution [2,3]. Other indications of a particularly dynamic evolution are the frequent mirror-image asymmetry in sister clades [4], re-appearance of symmetry within otherwise asymmetric clades [1,3,5] and cases of genetic polymorphism for direction of asymmetry [3,4,6].

Thanks to a wealth of information on genital morphology from the taxonomic literature [7], phylogenetic patterns are beginning to be elucidated [2,8,9]. However, a comprehensive understanding of the evolution of asymmetric genitalia has not yet emerged. Based on comparative evidence, Huber *et al.* [2] suggest that in insects, the repeated evolution from a plesiomorphic, female-above mating position to an apomorphic, male-dominated position, has led to male genital asymmetry via morphological accommodation of the required rotation and flexing of the abdominal tips. However, additional explanations are needed, as dramatic genital asymmetry appears and re-appears in many taxa (e.g. Coleoptera and Mammalia) that generally mate in an invariant, male-above position [3,9]. For such taxa, the pattern of genital asymmetry evolution appears to be a component of the more general phenomenon of rapid and divergent evolution in male genitalia [10], explained as the result of two not entirely mutually exclusive classes of sexual selection, namely cryptic female choice and sexually antagonistic co-evolution [7]. However, genital asymmetry has yet to emerge as a morphological phenomenon in its own right within this conceptual framework for genitalia evolution. In the context of male genital asymmetry, evolution under cryptic female choice would mean that, under some conditions, females prefer males with asymmetric genitalia—perhaps because decoupling of left and right sides of the genitalia allows a more varied mix of tactile signals. This may be why males with asymmetric claspers have higher fertilization success in the fly *Dryomyza anilis* [3,11]. Sexually antagonistic co-evolution, on the other hand, may be a possible explanation if male genital asymmetry were an adaptation to gaining control over asymmetric sperm deposition in the female (in the dung fly *Scatophaga stercoraria*, for example, females deposit sperm of favoured males preferentially in the right hand, paired spermathecae [12]).

**Figure 1.**
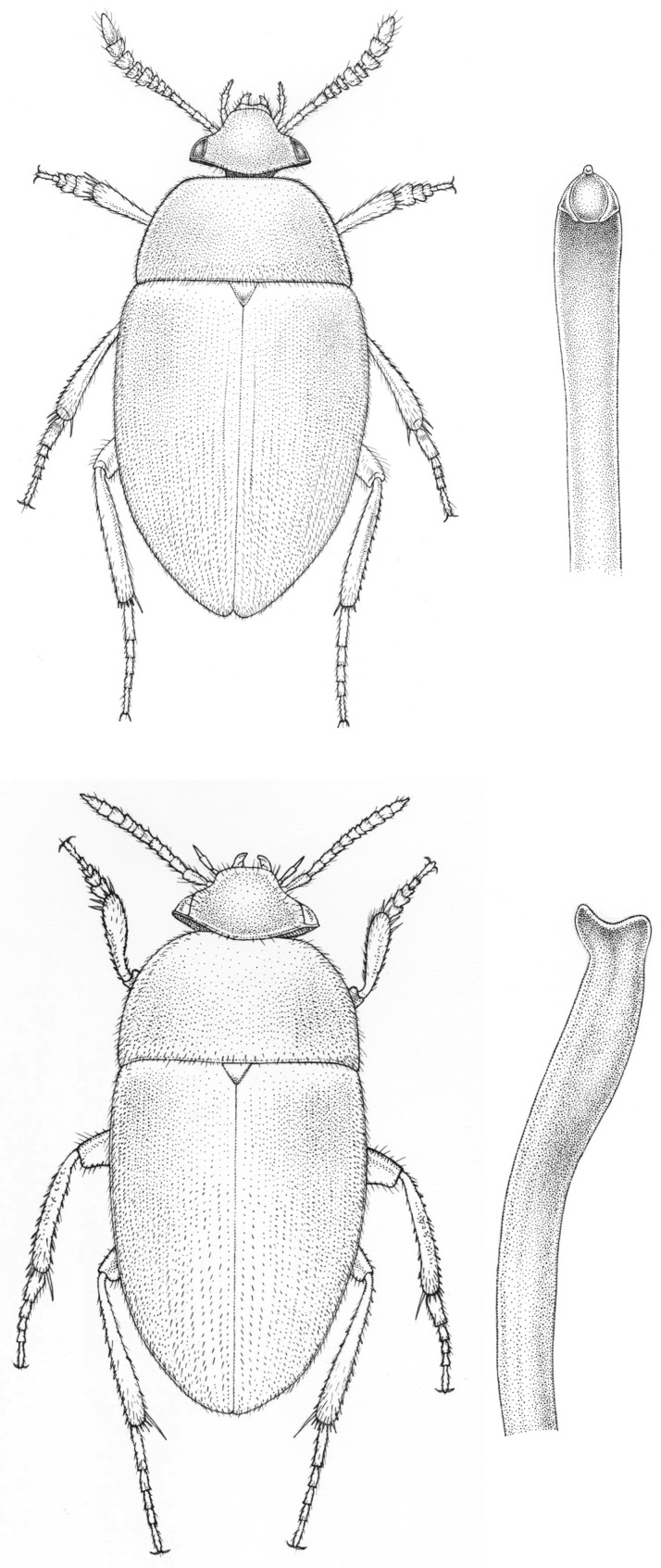
Intrageneric diversity in male genital symmetry. Shown are dorsal views of the habitus (left) and median lobe (right) of *Sciodrepoides watsoni* (top) and *S. fumatus* (bottom). Artwork by Erik-Jan Bosch (Naturalis Biodiversity Center).

It is unlikely that a single explanation will be found for a phenomenon in morphological evolution that stretches across the entire animal kingdom. Yet, we feel that genital asymmetry is such a widely distributed and recognizable pattern, that repeated studies in different taxa may eventually result in a broad understanding. In this paper, we explore aspects of genital asymmetry in beetles. In the Coleoptera, male genital asymmetry is very common, while mating positions are mostly symmetric, male-above. Informal perusal of the coleopterological literature [2,3,13], as well as two studies of scarabaeoid beetles at tribe and genus level [8,9] show repeated evolution of asymmetry in several components of the male, and in some cases [14] also the female, genitalia. Moreover, cases of chiral reversal and antisymmetry are available for coleopteran taxa that are easily cultured and studied in the laboratory [3,4,15], making Coleoptera a suitable group for further study of the ultimate causes for genital chirality. In this paper, we provide a set of observations on the evolution and development of asymmetric genitalia in beetles, which may serve as a basis for further work. We provide an informal assessment of symmetric and asymmetric male genitalia in all Coleoptera. We then analyse the evolution of asymmetry in various components of the male genital system separately in the tribe Cholevini (Leiodidae; figure 1). Finally, we provide suggestions for further experimental work.

## 2. Material and methods

### (a) Taxonomic distribution of genital asymmetry

We selected the comprehensive coleopteran infrafamilial phylogeny as presented in Hunt *et al.* [16] as the basis for a Coleoptera-wide assessment of male genital asymmetry. For 296 out of 335 higher level taxa (mostly families, groups of subfamilies, subfamilies or tribes) included in that tree, we obtained an indication of male genital asymmetry, based on perusal of 182 taxonomic works, mostly monographs, identification guides and taxonomic revisions. The data are presented in electronic supplementary material, S1. Information on endophallus and female genital (a)symmetry were very incomplete; therefore, we here refer only to the complex composed of the median lobe plus parameres, and class each taxon in one of five categories of (a)symmetry. These categories were: (i) always symmetric, (ii) mostly symmetric (a small proportion of species, *ca* 10% or less, asymmetric), (iii) symmetric/asymmetric (appreciable frequencies of both symmetry and asymmetry occur within the taxon), (iv) mostly asymmetric (a small proportion of species, *ca* 10% or less, symmetric) and (v) always asymmetric.

### (b) Molecular phylogenetics of Cholevini (Coleoptera: Leiodidae)

We obtained 191 Cholevini specimens belonging to 33 species. Specimens were collected in Europe, Japan and North America (electronic supplementary material, S2) and preserved in pure ethanol in the field. Additionally, we obtained published sequences from Fresneda *et al.* [17] and Schilthuizen *et al.* [18], and from GenBank, making a total of 212 individuals belonging to 40 nominal species. For the outgroup, we used one species each of four other Cholevinae tribes, namely *Graciliella apfelbecki* (Leptodirini), *Ptomaphagus subvillosus* (Ptomaphagini), *Speonemadus maroccanus* (Anemadini) and *Sciaphyes sibiricus* (Sciaphyini). The data for the former two species were generated in the present study, while those for the latter two were taken from Fresneda *et al.* [17]. From each specimen, we dissected the genitalia and removed three legs from one flank. The legs and sometimes also abdominal tissue were used for DNA extraction, whereas the body and the genitalia were mounted dry, pinned, labelled and stored in the Coleoptera collection of Naturalis Biodiversity Center. Each specimen was identified to species level. DNA extraction was done with a NucleoMag kit (Macherey-Nagel), and used in PCR reactions with primers and reaction conditions of Fresneda *et al.* [17] and Folmer *et al.* [19] to amplify three mitochondrial loci, namely the 5′ and 3′ halves of cytochrome *c* oxidase subunit 1 (which we here term *COIa* and *COIb*, respectively), an internal fragment of cytochrome *b* (*CytB*), a region consisting of the 3′ end of the large ribosomal unit 16S rDNA plus the leucine tRNA plus the 5′ end of NADH dehydrogenase subunit 1, (*rrnL* + *trnL* + *nad1*), and two nuclear loci, namely the 5′ end of the small ribosomal unit 18S rDNA (*18S*), and an internal fragment of the large ribosomal unit 28S rDNA (*28S*). PCR products were sequenced in both directions on an ABI 3730XL by Macrogen Corp.

We aligned all ingroup sequences either manually (for all mitochondrial loci, in which alignment was trivial) in BioEdit v. 7, or automatically (for the sequences with many indels, i.e. 18S and 28S) with MAFFT v. 7, as implemented on http://mafft.cbrc.jp/alignment/server/. We used the G-INS-i strategy, 20PAM/*k* = 2 scoring matrix, gap opening penalty of 1.53, and offset value of 0.1.

We first defined operational taxonomic units (OTUs) based on an all-specimen analysis for the 5′ and 3′ halves of *COI*, as well as *rrnL* *+* *trnL* *+* *nad1* (as intraspecific sequence variation in these mitochondrial loci tends to coalesce relatively fast, and also because we obtained these sequences for large numbers of specimens). This showed [20] that all taxonomically accepted species were clearly and unambiguously recovered, with the exception that the European and North American populations of *Sciodrepoides watsoni* and the Spanish and northern-European populations of *Nargus velox* showed up as separate OTUs, and that *Catops fuscus* and *C. nigricans* were inseparable—we, therefore, combined these latter two species into a single OTU. Consequently, our ingroup consisted of 41 OTUs.

We then reduced our dataset by merging all sequences for the same OTU in Mesquite v. 2.75 [21], to which we added the outgroup sequences. Merging all intra-OTU sequences introduces polymorphic sites. As MrBayes treats polymorphisms as uncertainties, this effectively means we remove intraspecific variation from the dataset, as recommended by Gittenberger *et al.* [22]. We then once again (for *18S*, *28S* and *rrnL* *+* *trnL* *+* *nad1*) aligned all sequence matrices (ingroup + outgroup) with MAFFT using the same settings as mentioned above. The alignment is available in TreeBase (http://purl.org/phylo/treebase/phylows/study/TB2:S19261). We selected a substitution and rate heterogeneity model for each locus in jModeltest 2 [23,24], setting the number of substitution schemes to three, to restrict the output to those models compatible with MrBayes. We used the Akaike information criterion with correction (AICc) for small sample size to evaluate the goodness-of-fit of the models. The substitution models chosen based on AICc were: GTR + I + *Γ* for *COIa*, *COIb*, *rrnL* *+* *trnL* *+* *nad1* and *28S*, GTR + *Γ* for *CytB*, and SYM + I + *Γ* for *18S*. We then used Mesquite to concatenate the data of all loci, producing a single data matrix of 5619 nucleotide positions (1–676, *COIa*; 677–1473, *rrnL* *+* *trnL* *+* *nad1*; 1474–3410, *18S*; 3411–4184, *28S*; 4185–5016, *COIb*; 5017–5619, *CytB*). We then ran a Bayesian analysis of 15 000 000 generations (after which the standard deviation of split frequencies had fallen below 0.01), while enforcing for each locus the respective molecular evolution model, and otherwise the same settings as above. Based on the phylogenetic reconstruction of Fresneda *et al.* [17,25], which resolve Leptodirini to be the sister group of all other Cholevinae, we used *G. apfelbecki* to root the tree.

### (c) Male genital morphology of Cholevini

The cholevine male genitalia consist of three chief components: (i) the two lateral, whip or drumstick-like parameres; the parameres are attached on either side of (ii) the median lobe, a hollow capsule that contains and directs (iii) the endophallus, a pliable, inflatable sac that, during copulation, protrudes via the door-like ligulae of the median lobe. The endophallus is often adorned with smaller or larger chitinous teeth or other sclerites. During copulation, the parameres and most of the median lobe remain outside of the female, whereas the tip of the median lobe is inserted into the genital segment, and the endophallus inflates into the vagina to deliver the ejaculate. The positioning of the endophallus appears to be modulated by the specific shape and degrees of flexibility of the median lobe and the ligulae [26]. For each of our OTUs, we dissected male genitalia of one or more individuals and inspected these via light and scanning-electron microscopy. Where possible, these were the same individuals or derived from the same populations as the individuals we used for the molecular work, but in many cases they were conspecifics that derived from the same general geographical area. In some cases, we used images from the literature (as available via http://cholevidae.myspecies.info) to complete our assessments. This yielded a two-state matrix for characters 1–4 as given above. The matrix is represented in table 1.

**Table 1.** Genital asymmetry in the studied OTUs of Cholevini.

OTU	parameres	median lobe	endophallus	major endophallus sclerite	sources
*Apocatops nigrita*	symmetric	symmetric	symmetric	symmetric	this study; [26]
*Catops americanus*	symmetric	symmetric	?	?	this study
*Catops andalusicus*	symmetric	symmetric	?	?	this study; [26]
*Catops angustitarsis lewisi*	symmetric	symmetric	symmetric	symmetric	this study; [27]
*Catops chrysomeloides*	symmetric	symmetric	?	?	this study
*Catops coracinus*	symmetric	symmetric	symmetric	symmetric	this study
*Catops fuliginosus*	symmetric	symmetric	symmetric	symmetric	this study
*Catops fuscus* + *nigricans*	symmetric	symmetric	symmetric	symmetric	this study
*Catops grandicollis*	symmetric	symmetric	symmetric	symmetric	this study
*Catops* cf. *hilleri*	symmetric	symmetric	?	?	this study; [27]
*Catops kirbyi*	symmetric	symmetric	?	?	this study
*Catops morio*	symmetric	symmetric	symmetric	symmetric	this study
*Catops neglectus*	symmetric	symmetric	symmetric	symmetric	this study
*Catops nigriclavis*	symmetric	symmetric	symmetric	symmetric	this study; [26]
*Catops picipes*	symmetric	symmetric	symmetric	symmetric	this study
*Catops subfuscus*	symmetric	symmetric	symmetric	symmetric	this study
*Catops tristis*	symmetric	symmetric	symmetric	symmetric	this study
*Choleva agilis*	symmetric	symmetric	symmetric	asymmetric	this study; [28]
*Choleva angustata*	symmetric	symmetric	symmetric	asymmetric	this study; [27]
*Choleva cisteloides*	symmetric	symmetric	symmetric	asymmetric	this study; [27,29]
*Choleva elongata*	symmetric	symmetric	symmetric	asymmetric	this study
*Choleva glauca*	symmetric	symmetric	symmetric	asymmetric	this study; [27,30]
*Choleva kocheri*	symmetric	symmetric	symmetric	asymmetric	this study; [31]
*Choleva oblonga*	symmetric	symmetric	symmetric	asymmetric	this study; [27]
*Choleva sturmi*	symmetric	symmetric	symmetric	asymmetric	this study; [27]
*Fissocatops westi*	symmetric	symmetric	symmetric	symmetric	this study; [26]
*Fusi nyujwa*	symmetric	symmetric	?	?	[32]
*Nargus algiricus*	symmetric	asymmetric	?	?	this study
*Nargus anisotomoides*	symmetric	symmetric	asymmetric	?	this study
*Nargus badius*	symmetric	asymmetric	?	?	this study
*Nargus brunneus*	symmetric	symmetric	?	?	this study
*Nargus velox* (Spain)	symmetric	asymmetric	?	?	this study
*Nargus velox* (N. Europe)	symmetric	asymmetric	asymmetric	asymmetric?	this study
*Nargus wilkini*	symmetric	symmetric	asymmetric	asymmetric	this study
*Prionochaeta harmandi*	symmetric	symmetric	?	?	this study
*Sciodrepoides fumatus*	symmetric	asymmetric	asymmetric	asymmetric	this study
*Sciodrepoides latinotum*	symmetric	symmetric	?	?	this study
*Sciodrepoides terminans*	symmetric	asymmetric	?	?	this study
*Sciodrepoides tsukamotoi*	symmetric	symmetric	symmetric	symmetric	this study
*Sciodrepoides watsoni* (Canada)	symmetric	symmetric	symmetric	symmetric	this study
*Sciodrepoides watsoni* (Europe)	symmetric	symmetric	symmetric	symmetric	this study
*Graciliella apfelbecki*	symmetric	symmetric	n.a.	n.a.	this study
*Ptomaphagus subvillosus*	symmetric	asymmetric	n.a.	asymmetric	this study
*Sciaphyes sibiricus*	symmetric	symmetric	?	?	this study; [17]
*Speonemadus maroccanus*	symmetric	symmetric	symmetric	symmetric	this study; [29]

## 3. Results

### (a) Phylogenetic distribution of genital asymmetry

Our perusal of the coleopteran literature showed that genital asymmetry in Coleoptera is present in at least 43% of the families we surveyed (77 out of 177; electronic supplementary material, S1). As already pointed out by Jeannel [13] and Huber *et al.* [2], this includes a wide variety of types of asymmetry. In some groups (e.g. Carabidae), the genital apparatus is rotated along the longitudinal axis, and the left and right parameres are shaped differently. In other groups, it is the median lobe that is asymmetric, but the asymmetry may be expressed on the proximal part (the basal orifice, as in Paussidae and Dryopidae) or the distal part (e.g. Ptomaphagini). In many taxa, the endophallus has not been sufficiently studied, but it appears that groups exist in which the endophallus is asymmetric even though the median lobe that encapsulates it is symmetric (e.g. Chelonariidae; see also [8]). Also, as observed previously [3], the distribution of asymmetry within a (sub)family may range from fixed across all species (as in, e.g. Mordellidae), via present in single genera or species groups (e.g. groups of *Phytodecta* species within the Chrysomelinae) to present in only single species (e.g. *Agathidium pilosum*, apparently the only species with asymmetric genitalia among the more than 2000 species of Leiodinae). Although a formal character evolution analysis on this dataset remains to be carried out, our results suggest that the distribution of male genital asymmetry is phylogenetically non-random, and seems to be much more rare among the large monophyletic infraorder Cucujiformia. In the Chrysomeloidea and Curculionoidea, for example, which jointly comprise one-third of all known beetle species [16], we recorded asymmetry in only 15 subfamilies, and in most of these it concerned isolated species or groups of species within genera.

### (b) Molecular phylogenetics of Cholevini (Coleoptera: Staphylinoidea: Leiodidae)

**Figure 2.**
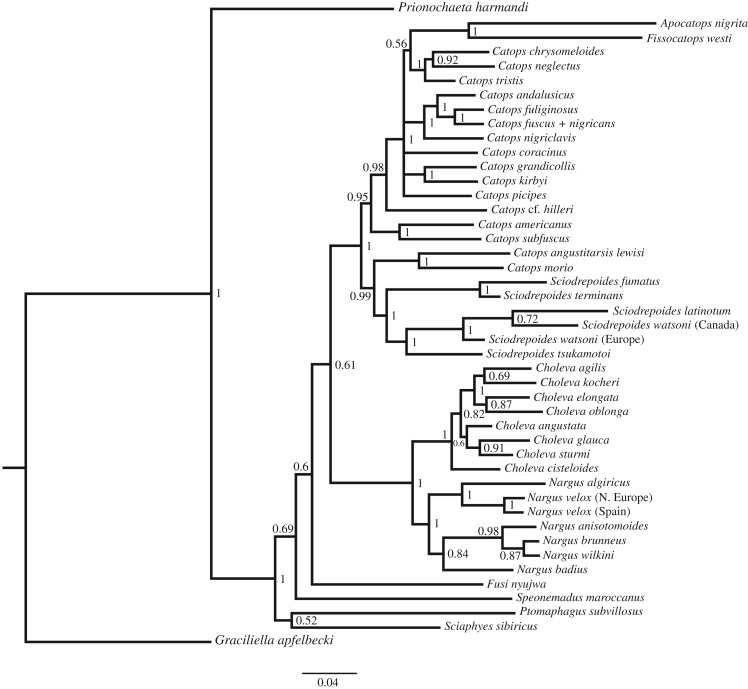
Phylogenetic reconstruction, based on four mitochondrial and two nuclear markers (total alignment: 5619 characters) of the Cholevini, and single representatives of each of four other Cholevinae tribes, namely *Graciliella apfelbecki* (Leptodirini), *Ptomaphagus subvillosus* (Ptomaphagini), *Speonemadus maroccanus* (Anemadini) and *Sciaphyes sibiricus* (Sciaphyini). The tree was rooted with *G. apfelbecki*. Numbers at the nodes are posterior probabilities in the Bayesian analysis.

Our phylogenetic analysis resulted in the reconstruction as shown in figure 2. We will discuss the taxonomic implications of the tree elsewhere, but briefly, the tree shows two main features. First, Cholevini as currently defined is not monophyletic: *Prionochaeta harmandi* is, unexpectedly, placed basally of three of the outgroup tribes. We, therefore, exclude this species for the ingroup character analysis below. Second, all genera are recovered as monophyletic groups, with the exception of *Catops*, which is paraphyletic with respect to *Sciodrepoides*, *Apocatops* and *Fissocatops*. However, the latter two genera may be incorrectly placed due to their long branches. In addition, we should point out that, due to unavailability of fresh samples for many taxa, our sampling of the Cholevini was very limited: the entire clade consists of 35 genera and subgenera, and more than 400 species (Perreau, i.l.). Genera with genital asymmetry exist that were excluded from our study, such as *Catoptrichus*, *Philomessor* and *Catopomorphus* [27].

### (c) Male genital morphology of Cholevini

We obtained (a)symmetry information for all OTUs for the parameres and median lobe. For the endophallus and its major sclerites, we failed to obtain information for a number of species, either because no specimens with inflated endophallus were available or because one or both characters were absent in the respective species (table 1).

The character analysis (using parsimony to reconstruct ancestral states) shows the following. First, all species had symmetric parameres. Second, asymmetry in the median lobe evolved twice (in the ancestor of *Nargus* and in the ancestor of the species pair *Sciodrepoides fumatus* + *S. terminans*), and has been lost once (in the ancestor of *N. anisotomoides*, *N. brunneus* and *N. wilkini*), or, alternatively, has been gained twice independently within *Nargus*. In the endophallus character, missing values prevent a detailed analysis, but we found an asymmetric endophallus in representatives of the entire genus *Nargus* (including the species with symmetric median lobe), and in *S. fumatus* (we could not obtain an everted endophallus for its sister species *S. terminans* which, like *S. fumatus*, also possesses an asymmetric median lobe). This suggests that endophallus asymmetry evolved twice. Finally, we found asymmetric major teeth on the endophallus in the same OTUs that had an asymmetric endophallus, but in addition also in all available representatives of *Choleva* (which has otherwise symmetric genitalia). This indicates that asymmetric major endophallus teeth also evolved twice, namely in the ancestor of *Nargus* + *Choleva* and also in the ancestor of *S. fumatus* + *S. terminans*.

## 4. Discussion

Although we have attempted to include a large proportion of beetle families, our overview of male genital asymmetry in the Coleoptera should not be seen as an end-point. First of all, our literature review was (unavoidably, for such a massive taxon as the beetles) very fragmentary and incomplete. For many subfamilies, we surveyed only a small fraction of the available taxonomic literature, and for some groups we failed to find any useful information on genital morphology at all. This means that more asymmetries will exist, even in groups that we record as fully symmetric. Also, we have not made a subdivision into asymmetries in different parts of the genitalia. Finally, information on female genital (a)symmetry was so rare (only in the taxonomy for a small number of families has there been a tradition to apply diagnostic female genital morphology) that we refrained from including this in our survey. For this and other reasons, we also refrained from a formal analysis to test general patterns in character evolution dynamics. Nonetheless, our tabulation allows for the formulation of one more specific hypothesis that may be tested with more extensive screening and analysis in the future. As mentioned above, the patterns suggest that asymmetry is rare within the Cucujiformia, a clade that contains more than half of all coleopteran species. As the Cucujiformia are best known for harbouring the most hyperdiverse herbivorous groups, phylogenetically independent contrasts may test the hypothesis that male genital asymmetry is associated with non-herbivory.

**Figure 3.**
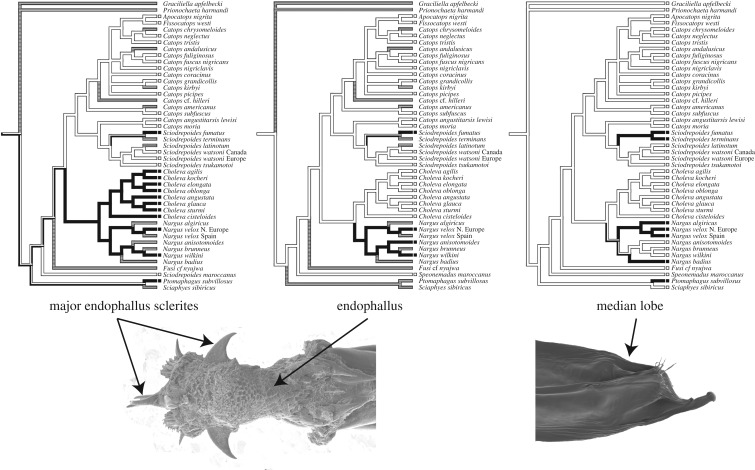
Genital (a)symmetry trait evolution reconstructed for the Cholevini tree of figure 2. Black lineages represent asymmetry, white lineages represent symmetry, and shaded lineages refer to uncertainty. Shown also are a dorsal view of the (symmetric) endophallus and its sclerites in *Sciodrepoides watsoni*, and ventral view of the (asymmetric) median lobe in *Nargus badius*.

Species-level phylogenies for smaller clades may provide more insights into the evolution of genital asymmetry, although these insights may not be generally applicable outside the taxon in question. In our sampling of the Cholevini, we found that asymmetry follows a partly nested pattern (figure 3). All OTUs, except those belonging to *Catops*, *Apocatops* and *Fissocatops*, had asymmetric major endophallus sclerites. A subset of these (all *Nargus* species, as well as *S. fumatus* and *S. terminans*) also had an asymmetric endophallus. Finally, an even smaller subset of these (a paraphyletic group within *Nargus* and the two *Sciodrepoides* species) had an asymmetric median lobe. None of the species had asymmetric parameres. This pattern suggests that asymmetry in the major teeth may be conditional to the evolution of asymmetry in the entire endophallus, and the latter again may be conditional to the evolution of an asymmetric median lobe. This in turn may imply that in some Cholevini species conditions exist that evoke a progressively asymmetric placement of the ejaculate by stronger asymmetric development of the supporting structures. By contrast, the parameres, which are generally considered structures for tactile stimulation of the female during copulation [10,33], were never asymmetric in the species we studied. This may indicate that in Cholevini, genital asymmetry chiefly evolves via the sexually antagonistic route. However, a much more comprehensive sampling of the Cholevini, as well as functional and experimental studies are needed to confirm this. For example, synchrotron micro-CT scanning of snap-frozen copulations (as we are currently performing in *Drosophila pachea*, a dipteran species with asymmetric male genitalia [34]) may reveal the effect of genital asymmetry on sperm transfer. Unlike Diptera, Coleoptera usually have a single unpaired spermatheca, but nevertheless there may be internal female asymmetries involved in female sperm storage control that the male's asymmetric sperm delivery apparatus may help to bypass. Further analyses of the anatomy of the female genitalia across multiple species are also required to obtain a better understanding of the pervasive but mysterious phenomenon of genital asymmetry and chirality.

## Supplementary Material

Supplementary File S1.

## Supplementary Material

Supplementary File S2.

## Data accessibility

The datasets supporting this article have been uploaded as part of the electronic supplementary material.
